# Targeting Macroautophagy as a Therapeutic Opportunity to Treat Parkinson’s Disease

**DOI:** 10.3389/fcell.2022.921314

**Published:** 2022-07-06

**Authors:** Irene Sanchez-Mirasierra, Saurav Ghimire, Sergio Hernandez-Diaz, Sandra-Fausia Soukup

**Affiliations:** Universite Bordeaux, CNRS, IMN, UMR 5293, F-33000 Bordeaux, France

**Keywords:** autophagy, Parkinson’s disease, autophagy modulators, PD causative proteins, potential therapeutic avenues

## Abstract

Macroautophagy, an evolutionary conserved catabolic process in the eukaryotic cell, regulates cellular homeostasis and plays a decisive role in self-engulfing proteins, protein aggregates, dysfunctional or damaged organelles, and invading pathogens. Growing evidence from *in vivo* and *in vitro* models shows that autophagy dysfunction plays decisive role in the pathogenesis of various neurodegenerative diseases, including Parkinson’s disease (PD). PD is an incurable and second most common neurodegenerative disease characterised by neurological and motor dysfunction accompanied of non-motor symptoms that can also reduce the life quality of patients. Despite the investment in research, the aetiology of the disease is still unknown and the therapies available are aimed mostly at ameliorating motor symptoms. Hence, therapeutics regulating the autophagy pathway might play an important role controlling the disease progression, reducing neuronal loss and even ameliorating non-motor symptoms. In this review, we highlight potential therapeutic opportunities involved in different targeting options like an initiation of autophagy, Leucine-rich repeat kinase 2 (LRRK2) inhibition, mitophagy, lysosomes, lipid metabolism, immune system, gene expression, biomarkers, and also non-pharmacological interventions. Thus, strategies to identify therapeutics targeting the pathways modulating autophagy might hold a future for therapy development against PD.

## Introduction

**FIGURE 1:**
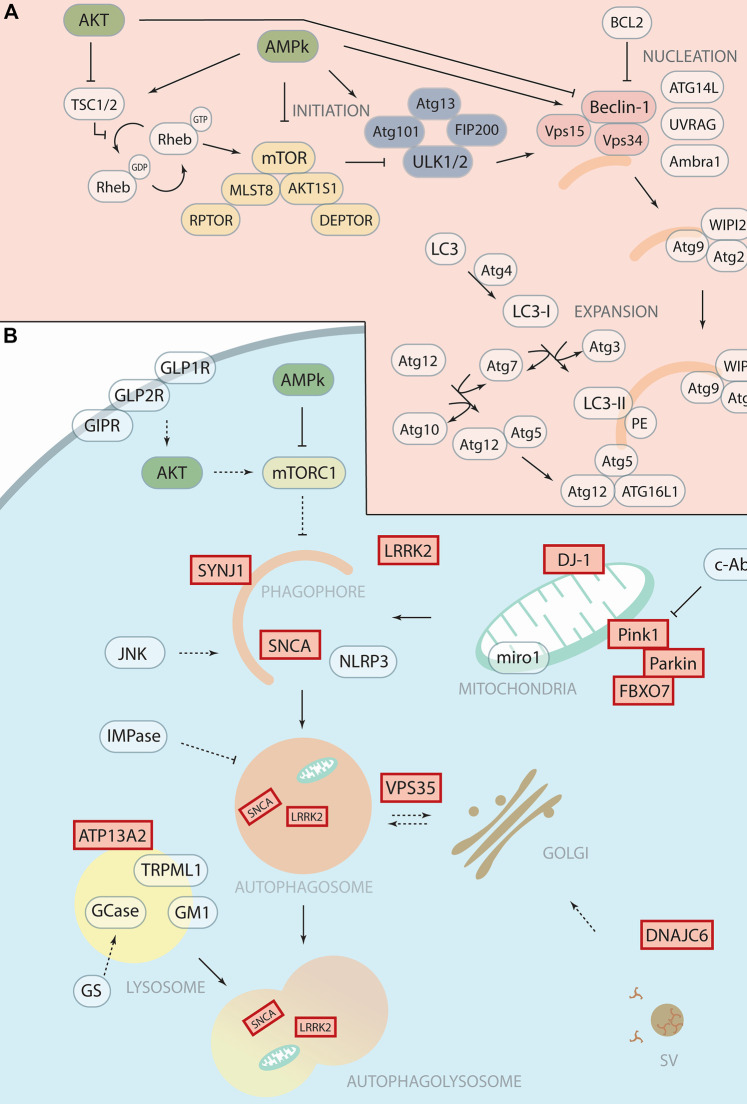
Therapeutic targeting of autophagy with potential application in Parkinson’s Disease. **(A)** Schematic representation of autophagosomal formation with autophagic core proteins involved in the different steps such as initiation, nucleation and expansion. We highlight in yellow the mTOR complex 1, in blue the ULK1 complex and in soft red the PI3K Class III complex. **(B)** Schematic representation of the autophagy pathway shows the forming phagophore that matures to the autophagosome and then fuses with the lysosome for degradation. Proteins encoded by “Parkinson’s disease genes,” labelled with red boxes, function in the autophagy pathway and related processes at different steps. The therapeutic targets or drugable nodes within the autophagy pathway that are targeted by compounds discussed in this minireview are marked in blue.

Macroautophagy (hereafter called autophagy) is an evolutionarily conserved catabolic process that regulates cellular homeostasis by recycling cytoplasmic components such as proteins, aggregates, damaged organelles, and pathogens. Autophagy consists of several steps (Figure 1A) for the engulfment of the cytosplasmic components into the autophagosome that in turn fuses to the lysosome, giving raise to the autolysosome where the lysosomal hydrolases degrade the target material (De Duve, 1963; Mari et al., 2011). Several studies show that alterations of autophagy are associated with neurodegeneration and various neurodegenerative diseases such as Alzheimer’s disease (AD), Amyotrophic lateral sclerosis (ALS), Huntington’s disease (HD) and Parkinson’s disease (PD) (Komatsu et al., 2006; Liang et al., 2010; Friedman et al., 2012) (Aman et al., 2021).

PD is the second most common neurodegenerative disorder, after AD, that affects mainly the dopaminergic (DA) neurons in the *substantia nigra pars compacta* (SNc). PD is characterised by three cardinal motor symptoms, akinesia/bradykinesia, tremor at rest and rigidity, in combination with other non-motor symptoms. Currently, there is no known cure or treatment to stop the progression of the disease. PD is often diagnosed when motor symptoms appear, and at this stage around 30%–70% of the DA neurons in the SNc (Fearnley and Lees, 1991; Cheng et al., 2010; Giguère et al., 2018) has already degenerated. Interestingly, various PD causative proteins have a direct role in autophagy, such as LRRK2, Synaptojanin1, Pink1, and Parkin (Soukup et al., 2016; Vanhauwaert et al., 2017) (Figure 1B). Another PD hallmark often associated with the loss of DA neurons is SNCA (α-Synuclein) aggregation (Soukup et al., 2018) that results from either mutations in SNCA or an overabundance of the protein. Although the resulting aggregates can be degraded by autophagy (Vogiatzi et al., 2008) SNCA can also impair autophagic protein degradation in PD while autophagy inhibition seems to exacerbate SNCA accumulation (Lei et al., 2019) (Song et al., 2014) (Klucken et al., 2012). Genome Wide Association Studies (GWAS) and post-mortem analysis linked various autophagy associated genes as risk factors in PD (Anglade et al., 1997; Chen et al., 2013; Mamais et al., 2018).

This review recapitulates the molecular mechanisms of macroautophagy in PD, focusing on recent clinical studies seeking to exploit autophagy to halt the progression of PD. Finally, we discuss the current gap and future perspective of pharmacological and non-pharmaceutical approaches that modulate autophagy as a therapeutic avenue to treat PD.

## Targeting the Initiation of Autophagy

Amino acid starvation ultimately leads to mTOR phosphorylation and inactivation, promoting autophagy. Similarly, changes in cellular energy levels (AMP/ATP ratio) or glucose starvation can activate AMP-activated protein kinase (PRKAA1*/*AMPK), leading to the inactivation of mTOR, activation of ULK1, and initiation of autophagy. Conversely, the AKT pathway can negatively regulate autophagy and is often deregulated in neurodegenerative disorders (Long et al., 2021). Thus, therapeutical strategies could exploit metabolic pathways like AMPK, AKT, or mTOR to control autophagy. However, targeting these proteins produce widespread off-target effects (Xiang et al., 2020). For example, the phosphodiesterase inhibitor ibudilast inhibits mTOR complex 1 and promotes the nuclear translocation of transcription factor EB resulting in higher transcription of autophagy genes that induces lysosomal and autophagy biogenesis (Chen et al., 2020). The flavonoid Icariin restore the levels of autophagy-related proteins (LC3-II, beclin1, p62, and mTOR) and has shown to be neuroprotective, in rotenone induced rat PD model and PC12 cells (Zeng et al., 2019; Wang S. et al., 2021). Further, combinations of drugs like the mTOR allosteric-inhibitor sirolimus (also known as rapamycin) with RTB101 (ATP-competitor that also inhibits mTOR) that crosses the blood-brain barrier (BBB) in concentrations capable of inducing autophagy, may provide novel potential approaches in PD therapy (GlobeNewsWire, 2020). However, compounds such as Trehalose that promote autophagy *via* mTOR-independent pathway are also emerging as a promising therapeutic candidates for treating PD (Khalifeh et al., 2019).

Currently, symptomatic PD treatment relies on a dopamine replacement therapy consisting of levodopa (L-Dopa) administration. Unfortunately, this treatment causes several problems including motor fluctuations and abnormal involuntary movements named dyskinesia; although the mechanism is still unclear, a recent study reported that the administration of rapamycin effectively reduced this L-dopa-induced dyskinesia (Feyder et al., 2021). Glucagon-like peptide 1 receptor (GLP1R) that indirectly targets AKT might help as an adjuvant therapy. For example, the Glucagon-like peptide 1 analogue Liraglutide reduces the adverse side effects of L-DOPA administration (Badawi et al., 2019) and also enhances mitophagy flux (Lin et al., 2021). New approaches to restore autophagy, such as dual formulation with GLPR agonists (DA-CH5) (Zhang et al., 2020) or GLP2R analogues (Su Y. et al., 2021), are being explored.

Drugs modulating the neuroprotective AMPK pathway are also being tested. For example, the anti-diabetic drug Metformin triggers autophagy by activating AMPK and increases the tyrosine hydroxylase-stained neurons in the SN in rotenone induced mouse PD model (El-Ghaiesh et al., 2020; Katila et al., 2020). Unfortunately, this drug was associated with an increased PD development risk (Ping et al., 2020; Qin et al., 2021). The polyphenol Resveratrol can induce autophagy by mTOR inhibition and shows neuroprotection in MPTP (Su C.-F. et al., 2021), rotenone, 6-OHDA, paraquat, maneb induced and transgenic mouse PD model (Liu et al., 2019) but whether this drug acts *via* AMPK or PI3K/AKT is still unclear (Arbo et al., 2020). Non-pharmacological therapeutic approaches targeting energy metabolism, such as the ketone diet, improved motor (Norwitz et al., 2020) and cognitive (Krikorian et al., 2019) performances in PD patients and the ketone βHB can act through HCAR2, activating AMPK and therefore autophagy (Kovács et al., 2021). Overall, exercise seems to demonstrate neuroprotection in PD patients (Yang et al., 2021a), and restore autophagy markers (Ferreira et al., 2021) but additional research is necessary to explore the cellular circuits and molecular mechanisms underlying the intersection between autophagy and exercise leading to neuroprotection.

## Leucine-Rich Repeat Kinase 2 Inhibition

LRRK2 is a multifunctional protein with threonine and serine kinase domains that genetically contributes to PD (Paisán-Ruíz et al., 2004). Various studies reported a context-dependent function of LRRK2 in several cellular processes such as autophagy and lysosomal degradation depending on the specific LRRK2 mutation, cell type or even in the step of the autophagy (Madureira et al., 2020) (Wallings et al., 2015). At the presynaptic terminal, LRRK2 phosphorylates Endophilin-A to attract autophagic proteins like Atg3, thus initiating autophagy induction (Soukup et al., 2016). The Endophilin-A LRRK2 interaction seems to function upstream of the autophagic protein Endophilin-B in autophagy induction (Hernandez-Diaz et al., 2022). This is particularly interesting since Histone deacetylase 2 (HDAC2) regulates neuronal Endophilin-B1 expression (Wang D. B. et al., 2019) and could be a promising therapeutic target to restore impaired autophagy in PD patients (Mazzocchi et al., 2020). Indeed, LRRK2 kinase gain-of-function mutations, such as G2019S, are the most common cause of familial PD and activation of LRRK2 has also been reported in sporadic PD (Maio et al., 2018). Therefore, the inhibition of LRRK2 kinase activity is being explored as a potential therapy (Ding and Ren, 2020) and reducing LRRK2 activity in animal models of PD is reported to minimise SNCA aggregation, neuroinflammation, and dopaminergic neuron loss (Daher et al., 2014) (Daher et al., 2015). An example of this strategy is the inhibitor DNL151, proceeding into later clinical phases due to its dosage flexibility and ability to cross BBB (Denali Therapeutics Inc, 2020). Antisense oligonucleotides constitute an alternative approach to target LRRK2. Intracerebral injection of antisense oligonucleotides in a PD mouse model reduced LRRK2 protein levels as well as SNCA aggregation and dopaminergic neurodegeneration (Zhao et al., 2017). Indeed, intrathecal injection of the drug BIIB094, an antisense oligonucleotide that targets LRRK2 mRNA for degradation, is currently under clinical phase 1 (Supplementary Table S1).

## Mitophagy

Mitochondria plays an important role in the pathogenesis of PD (González-Rodríguez et al., 2021) (Sanchez-mirasierra et al., 2021) (Cen et al., 2021). Mitophagy is the selective autophagy mechanism that targets mitochondria to the autophagy pathway (Lemasters, 2005) *via* specific mitophagy receptors such as optineurin (OPTN) or nuclear dot protein 52 kDa (NDP52) (Lazarou et al., 2015), (Heo et al., 2015). There are Pink1/Parkin dependent and independent mechanisms of mitophagy. PINK1 and PRKN act sequentially in mitophagy to target damaged mitochondria for degradation (Narendra et al., 2008) (Narendra et al., 2010). While Parkin/Pink1 may play additional roles in mitochondrial fission and fussion events (Deng et al., 2008), loss-of-function mutation in Parkin and PINK1 are linked to early-onset PD [(Kitada et al., 1998) (Valente et al., 2001)]. Targeting PARKIN activity regulating posttranscriptional modifications, such as ULK1 phosphorilation of PARKIN (Hung et al., 2021) or directly controlling mitochondrial fission and fusion (Deng et al., 2008) emerge as therapeutic opportunities in PD. However, most of the studies involving the role of PINK1 and PARKIN in mitophagy have been done *in vitro*. Experimental *in vivo* evidence provides a more complex picture showing that the role of PARKIN-dependent mitophagy is restricted to the cell soma and not to axonal or other distal compartments (Sung et al., 2016). Moreover, several *in vivo* studies report tissue-specific differences in the levels of basal mitophagy (Sun et al., 2015) (McWilliams et al., 2016) or even showing that basal mitophagy can occur independently of PINK1 (McWilliams et al., 2018). This evidence demonstrates the importance of context and cell specificity in mitophagy and should be taken into account for therapeutic approaches. In PINK1-PARKIN-independent mitophagy, mitochondrial proteins interact directly with the autophagic LC3/GABARAP family proteins (Antón et al., 2016). For example, genetic and pharmacological induction of the mitochondrial receptor NIX restores mitophagy independently of PINK1/PARKIN in PD patients cells (Koentjoro et al., 2017). Unfortunately, the molecular mechanisms of mitochondrial receptors underlying mitophagy are not well understood *in vivo*. Nevertheless, therapeutics targeting mitophagy are a promising approach. For instance, celastrol promotes DA neuron survival in mice PD models (Zhang C. et al., 2021) (Lin et al., 2019) and suppresses PARKIN recruitment to the mitochondria by inactivating PINK1 (Zhang et al., 2019). An alternative therapeutic target is the protein Miro1, a mitochondrial protein, that is, a risk factor (Grossmann et al., 2020) and a biomarker of PD (Hsieh et al., 2019) even in the prodromal phase (Nguyen et al., 2021). The accumulation of this protein delays mitophagy (Shaltouki et al., 2018) and the R272Q mutation in the Miro1 protein blocks autophagy flux (Berenguer-escuder et al., 2020). However, evidence also suggests that complete loss of Miro1 disrupts mitophagy and induces a stress response (Lopez-Domenech et al., 2021).

## Targeting Lysosomes

At the latest step of autophagy, the autophagosome fuses with the acidic lysosome raising the autolysosome, where degradation occurs. Mutations in ATP13A2 gene, which encodes a lysosomal P5-type ATPase, leads to parkinsonism (Ramirez et al., 2006) and impaired lysosomal acidification, thus decreasing lysosome mediated clearance of autophagosomes (Dehay et al., 2012). Therefore, enhancing the degradation step of autophagy emerged as a therapeutic strategy. The drugs like rifampicin (Liang et al., 2020) and clioquinol (Wallings et al., 2019) (Shi et al., 2020) are promising candidates for restoring lysosomal acidification in mammalian models of PD. The compound ML-SA1 reduces SNCA accumulation in DA neurons from PD patients by increasing lysosomal biogenesis and function (Tedeschi et al., 2019; Tsunemi et al., 2019). Targeting lysosomal function currently has an ample clinical pipeline: the drug Ambroxol is currently approved for the treatment of respiratory diseases, but preclinical and clinical data support a therapeutic potential for PD patients (Silveira et al., 2019; Mullin et al., 2020; Istaiti et al., 2021). These findings strongly support the hypothesis that dysfunction of the lysosome has a pivotal role in the etiology of PD. Hence, drugs targeting the lysosomal pathway might prevent neuronal death and neuronal function, thereby halting PD progression.

Other pharmacological alternatives focus on the inhibition of c-Abl, a kinase that functions in the late stage of the autophagy pathway by regulating lysosome maturation *via* the lysosomal enzymes cathepsin D and cathepsin L (Yogalingam and Pendergast, 2008). Activation of this protein causes autophagy deficits and accumulation of SNCA, resulting in DA neurodegeneration (Karim et al., 2020). The c-Abl modulator, Nilotinib, has a safety profile but showed no improvement in motor scores and whether this candidate will further proceed to phase 3 in clinical trials is unclear (Pagan et al., 2020; Simuni et al., 2021).

## Targeting Lipid Metabolism

Emerging evidence shows that deregulation of lipid metabolism can ultimately play a role in the onset and progression of PD (Hernandez-Diaz and Soukup, 2020). Several lipid families (fatty acyls, glycerolipids, glycerophospholipids, sphingolipids, and sterols) have been so far identified as regulators of the PD aetiological processes such as SNCA aggregation, oxidative- and endoplasmic reticulum-stress, endosomal-lysosomal dysfunction, and immune response (Xicoy et al., 2019b). Moreover, several enzymes involved in lipid metabolism can contribute directly or indirectly to the mechanism of PD progression (Alecu and Bennett, 2019). Therefore, molecules targeting those enzymes, lipid species and specific lipid pathways might interfere with the disease progression. For instance, GWAS indicates that the gene Glucocerebrosidase1 (GBA1) is a risk factor for PD. Mutations in GBA1 are present in about 10% of all patients with sporadic PD worldwide (Avenali et al., 2020). GBA1 encodes for the lysosomal enzyme glucocerebrosidase (GCase), which catalyses the hydrolysis of glucosylceramide to ceramide and glucose. GCase mutations can decrease GCase function, impairing Ceramide (Cer) production, synthesis and recycling of sphingolipids (Wigger et al., 2019) and also promoting the accumulation of the GCase substrate glucosylceramide. Changes in Cer, glucosylceramide, cholesterol and different sphingolipid species can modulate lipid membrane morphology (Akiyama et al., 2013) (García-Sanz et al., 2021), autophagy at different levels (Hernandez-Diaz and Soukup, 2020) and even the levels of lysosomal cholesterol could contribute to change in lipid rafts that are necessary for synaptic function and integrity (García-Sanz et al., 2021). Mutations in GBA1 and glucosylceramide accumulation can impair lysosomal function, mitochondrial alterations, endoplasmic reticulum stress, and abnormal accumulation of SNCA (Avenali et al., 2020) (Farfel-Becker et al., 2014). Indeed, drugs targeting mutant GBA1 could reduce the progression of PD by halting the activity of glucocerebrosidase and increasing activity in cellular and mice models along with a reduction in SNCA accumulation (Fernandes et al., 2016; Parnetti et al., 2017). Currently, several compounds targeting GBA (PR001, Ambroxol, venglustat GZ/SAR402671) are in various phases of clinical trials (Mullin et al., 2020) (Supplementary Table S1).

Gangliosides are glycosphingolipids that contain a sialic acid group. These oligosaccharides are important components of the neuronal plasma membranes. Accumulating evidence supports the role of gangliosides in the onset and progression of PD (Alecu and Bennett, 2019). In particular, experiments in animal PD models have demonstrated that gangliosides (GM1 and GM2) partially restore depleted dopamine levels, rescue motor symptoms, restore neurotransmitter levels, reduce SNCA aggregation and promote neuronal recovery (Schneider et al., 1995; Chiricozzi et al., 2019). In line with those findings, gangliosides or Ganglioside analogues (LIGA-20) intake promotes neuronal and motor function recovery (Alecu and Bennett, 2019). Thus, understanding ganglioside metabolism and neuroprotective roles seem essential to design novel therapeutic approaches. A recent study reported that PD patients exhibit lower serum levels of total cholesterol (TC), low density lipoprotein-cholesterol (LDL-C), and triglycerides (TG) compared to healthy individuals (Saedi et al., 2021). Further *in vitro* studies have demonstrated that change in the lipid profile is associated with PD causative agents (i.e., oxidative stress, endosomal-lysosomal function, endoplasmic reticulum stress, and immune response), and thus in PD etiology (Alecu and Bennett, 2019). Taken together these results highlights a strong correlation between abnormalities in lipid metabolism and PD. How these lipid alterations impact the lipid composition of the autophagosome structures is still unknown (Hernandez-Diaz and Soukup, 2020). Moreover, there is limited knowledge regarding the lipid profiles of the autophagic structures (Laczkó-Dobos et al., 2021) and a precise characterisation of the lipid composition of autophagosomal membranes in brain cells is currently missing. Taken together, the development of specific and safe drugs targeting lipid metabolism might open new avenues for treatments in PD.

## Immune System

The immune system may be overactive in PD and preclinical studies have further enlightened that neuroinflammation is associated with T cell infiltration, Lewy body formation, and microglia activation, which intensify the neuronal dysfunction by generating a detrimental immune response (Kannarkat et al., 2013; von Euler Chelpin and Vorup-Jensen, 2017). Further, studies reported that inflammation causes loss of DA neurons and how altered immune response could influence the onset and progression of neurodegenerative diseases, particularly PD (Tansey and Goldberg, 2010; Kannarkat et al., 2013; Chao et al., 2014). Currently several studies provide evidence supporting a correlation between blood brain barrier (BBB) disruption, DA neuronal cell death and PD (Rite et al., 2007) (Al-Bachari et al., 2020) Positron emission tomography (PET) and histological studies in patients with PD demonstrated dysfunction of the BBB system, alteration of the blood vessels and neurodegeneration in the SNc (Kortekaas et al., 2005) (Gray and Woulfe, 2015). In summary, these studies indicate that neuroinflammatory changes affect BBB dysfunction by altering transport systems, enhancing immune cell entry thereby enhancing PD pathogenesis and progression. There are some connections between inflammation, autophagy and DA neuronal loss. For example, in mice, loss of the autophagic core protein Atg5 leads to the activation of the inflammasome in microglia, leading to the protection of DA neurons and the recapitulation of some PD motor symptoms (Wang X. et al., 2019) Following cellular damage or infection, the inflammasome, a multimeric protein complex, is recruited by NOD-like receptor protein 3 (NLRP3) and SNCA oligomers mediate the activation of the NLRP3-inflammasome in PD by upregulating the expression of the autophagy-related protein ATG5 (Wang X. et al., 2019). Indeed, pharmacological inhibition of NLRP3 in mice lacking ATG5 in microglia rescues DA neuron loss (Cheng et al., 2020).

LRRK2 protein is expressed in the nervous system and immune cells, especially in response to inflammatory signals [reviewed in (Wallings and Tansey, 2019)]. Strikingly, growing evidence supports the role of LRRK2 in inflammation and the onset of inflammatory diseases such as Crohn’s disease, an inflammatory Bowel disease (IBD) (Hui et al., 2018) and patients with IBD are more likely to develop PD (Villumsen et al., 2019). However, further research is needed to understand how LRRK2 regulates immune pathways and whether current and new drugs targeting LRRK2 may also target the abnormal inflammation seen in PD patients.

Several studies have shown that active (boosting self-generate antibodies) or passive (administration of antibodies externally) immunotherapies could halt PD progression. For instance, using specific antibodies against toxic variants of SNCA can slow the progression of the disease (Lee and Lee, 2016; Oliveira et al., 2021). For instance, the injection of immunomodulatory growth factors like Sargramostim (GM-CSF Leukine) may protect DA neurons, resulting in the reduction of motor symptoms (Gendelman et al., 2017). However, the interactions between neuronal dysfunction, innate immune activation and neuroinflammation are still not well understood. Further research in this area is necessary to improve therapeutic approaches to alleviate motor and cognitive behaviours in PD.

## Targeting Gene Expression

Intensive research based on GWAS has identified that 5%–10% of individuals suffering from PD show dominant or recessive mutations in specific autophagy-related genes such as LRRK2, VPS35, DNAJC6; PARKIN, ATP13A2/Park9 or PINK1/Park6 (Satake et al., 2009; Simón-Sánchez et al., 2009; Klein and Westenberger, 2012). Hence, efforts to treat PD using gene therapy could restore pathophysiological pathways, halting neurodegeneration. This approach could compensate for the DA signalling in the basal ganglia, thereby providing neuroprotective effects and rescuing motor symptoms (Axelsen and Woldbye, 2018).

Recent *in vivo* and *in vitro* studies have shown that drugs targeting transcription factors (TFs) that regulate autophagy and lysosomal functions can be a promising strategy to prevent neurodegeneration. Transcription factor EB (TFEB) is a master regulator of lysosomal genes (Sardiello et al., 2009) and modulating TFEB activity is proposed as a therapeutic approach to treat PD pathogenesis (Decressac et al., 2013) and consequently there are a number of TFEB agonists being tested in preclinical trials (Chen et al., 2021). An additional therapeutic candidate is the TFs family FoxO, which transactivate genes that control autophagosome biogenesis and the formation of the autolysosome. Regulation of FoxO activity constitute a promising therapeutic strategy in the fields of senescence and age-related diseases (Calissi et al., 2021) which could potentially benefit the treatment of PD For instance, targeting the expression of the transcription factor FoxO6 (Desai et al., 2020) and FoxO3 (Pino et al., 2014) emerges as a promising alternative to regulate autophagy and prevent neurodegeneration by promoting the removal of misfolded, aggregated, toxic proteins (Dumitriu et al., 2012; Santo and Paik, 2018). Overall, based on the preclinical studies, it is clear that approaches targeting gene expression can effectively treat PD. However, further studies are essential to understand the pathways and signals playing a pivotal role in the onset and progression of PD.

## Biomarkers

One of the major hindrances to the development of a therapy for PD is the long latency period between the onset of degeneration of DA neurons and the manifestation of clinical symptoms (Schrag et al., 2017; Li and Le, 2020; Santaella et al., 2020). Biomarkers can accurately make risk predictions, prognostic, staging, theragnostic, and response evaluation of the disease years before the manifestation of major clinical features (Xicoy et al., 2019a; Shao and Le, 2019; Lee et al., 2021). Hence, identifying early predictive and prognostic PD biomarkers is essential for monitoring disease progression and evaluating of the positive response of the therapeutic intervention. Dysregulation of autophagy occurs in PD and biomarkers monitoring autophagy or autophagy-associated proteins will be valuable in managing PD (Zhang K. et al., 2021). For instance, a study has shown that the autophagy protein LC3B in cerebrospinal fluid can be a potential biomarker for early diagnosis of PD (Youn et al., 2018). In recent years, microRNAs (miRNAs) and long non-coding RNAs (lncRNAs) have emerged as effective biomarkers for the onset and progression of the disease. Several miRNAs (e.g., MiR124, MiR7, MiR153, miR253, and miR223) are under evaluation as diagnostic or prognostic biomarkers for cancer, heart disorders and also neurodegenerative diseases as PD (Ma et al., 2018; Angelopoulou et al., 2019; Akkoc and Gozuacik, 2020; Yang et al., 2021b). Moreover, biomarkers for the activity of autophagy could also provide information concerning the autophagy-related endosomal, lysosomal and secretary pathways; synucleinopathy processes, axonal damage biomarkers and even inherited PD-related mutations (DJ-1, LRRK2) (Youn et al.; Pan et al., 2008; Xicoy et al., 2019a).

Hence, identifying potential biomarkers associated with autophagy for the early diagnosis, prognosis and tracking the severity of disease could be essential for developing effective neuroprotective treatment strategies.

## Discussion

Currently available treatments of PD are based on symptomatic relief but neither restore lost neurons nor prevent or stop neurodegeneration. A rapid way to implement new therapies is through drug repurposing, which allows previously approved drugs to go directly into phase 2, shortening the length and cost of clinical trials as they already have a safety track record. In the pipeline of drug candidates to treat PD, at least 16 compounds have that characteristic (Stott et al., 2021), including drugs targeting proteins linked to autophagy, such as quetiapine, an antipsychotic that targets GCase (Burbulla et al., 2021). However, drug repurposing can be challenging (Keerie et al., 2021) and the identification of novel autophagy targets [such as ubiquitin (Schmidt et al., 2021)] and new drugs targeting this process is of utmost importance to use autophagy as a therapeutic tool in PD (Stacchiotti and Corsetti, 2020; Wang Z.-Y. et al., 2021; Li et al., 2021). Supplementary Table S1 enlists the pharmaceutical modulators of autophagy discussed in this minireview.

Medical chemistry can also help find novel treatments by optimising formulations to improve some characteristics of the existing compounds. For example, this approach has been used to enhance the anti-parkinsonian efficacy of resveratrol (Xiong et al., 2020), to increase the neuroprotective effect of metformin by the upregulation of autophagy (Agostini et al., 2022) and to improve mitochondrial function (Schlichtmann et al., 2021). Targeting the brain is still a challenge for any pharmacological approach. Thus, further investigations of new ways of encapsulation and administration would be essential for valid treatments (Ribeiro et al., 2020; Cunha et al., 2021). A promising way of delivery is through exosomes (Luo et al., 2021). However, the interplay between autophagy and exosome release and uptake needs to be further investigated at a cellular level, especially in the context of PD.

PD is currently considered a multifactorial and heterogeneous disease and novel classifications using PD subtypes are emerging to improve treatments and design better clinical trials. Indeed, this heterogeneity can impact drug treatment. For instance, the different mutations identified in LRRK2 gene produce neuropathological variations and the outcome of using LRRK2 inhibitors, which will differ in binding the open or the close conformation to produce different phosphorylated substrates making one mutation resistant to one type of inhibitor but not the other (Tasegian et al., 2021). Moreover, among the mutations involving GBA1, there are also null mutations that will require a replacement therapy and will not benefit from an activator (von Linstow et al., 2020). A precision medicine approach (using current knowledge of genetic risk in combination with genetic information and biomarkers from individual patients) will help to solve some of the current challenges in the design of clinical trials and therapies for PD patients (Schneider et al., 2020).

Another challenge to design trials to evaluate neuroprotection is how to select the patients in the prodromal phase (before developing motor symptoms) of PD. For example, accumulation of Miro1 occurs in PD patients (sporadic or associated with mutations) whereas, in healthy conditions, this protein is rapidly cleared in response to mitochondria damage, allowing the initiation of mitophagy. Thus, the clearance of Miro1 is proposed as a test to improve the success of clinical trials (Bharat and Wang, 2020). Another criterion that has been used with a metformin trial is to check its neuroprotection property in patients with idiopathic rapid eye movement behaviour disorder (iRBD). Patients with iRBD might develop PD, dementia with Lewy bodies or multiple system atrophy in 80% or more cases (Sportelli et al., 2020). To act effectively, we need markers for the onset and the progression of the disease. For example, macrophages from GBA-PD were successfully used to assess ambroxol therapy (Kopytova et al., 2021). Herein, Figure 1B we have discussed the drugable nodes as well as the known-genes implicated in the autophagic process and PD that has been discussed in this minireview.

Further investigations are needed to delimit autophagy therapeutical potential because of the dual face of autophagy mediating survival and cell death (Lamb, 2020). Hence understanding how autophagy proteins participate in other pathways is necessary to anticipate and minimise noxious off-target effects upon targeting these proteins (Xiang et al., 2020). Pharmacological efforts need to be made to improve organ, cell (neuron, glia) or even compartment specificity (soma, axon, synapse) of the drugs, as the effects of autophagy activation and inhibition can be context specific. Besides, non-pharmacological approaches to modulate autophagy should be explored, such as exercise or diet. In conclusion, autophagy represents a great therapeutic opportunity to treat PD; yet the details of the molecular mechanisms governing autophagy in different brain cells and compounds targeting it, as well as the crosstalk between autophagy and other pathways need to be elucidated, to unlock the therapeutic potential to fight against PD in the near future.
