# Supplementary material for: Targeting Macroautophagy as a Therapeutic Opportunity to Treat Parkinson’s Disease
Source: Front Cell Dev Biol. 2022 Jul 6;10:921314. doi: 10.3389/fcell.2022.921314 (PMC9298504; doi:10.3389/fcell.2022.921314)
Supplement: Supplementary file 1 [file Table1.pdf]

Target	Modulator	Mechanism of action	Stage/Phase of trial	Reference
AMPK	Exercise	• Upregulates CAMKIIa expression level.<br>• Promotes the expression of autophagy markers (beclin1 and LC3B).	Phase III<br>(2019-2024)<br>(2019-2024)	[1]<br>NCT03808675<br>NCT04000360
	Ganoderma lucidum*	• Stimulates autophagy, decreases LC3II/LC3I.<br>• Reverse alterations of AMPK, mTOR and ULK, PINK and PARKIN.	Phase III<br>2018-2021	[2]<br>NCT03594656
	Ketone bodies	• Improves mitochondrial function.<br>• Functions through βHB/HCAR2/AMPK/SIRT1-3/PGC1-α pathways.	Phase N/A<br>(2019-2021)<br>2021	[3][4]<br>NCT04477161<br>NCT04322461
	Metformin	• Reduces cell death.<br>• Higher expression of Nrf2, HO-1, AMPK, FOXO3 and thioredoxin.<br>• Inhibits VEGF.<br>• Involves in AMPK/aPKCζ/CREB pathway.	Phase III<br>(2018-2020)	NCT03685357
			Rotenone mice	[5]
			Preclinical: Mice and SH-SY5Y cell	[6]
	miR124	• Helps in the miRNA assay for the early diagnosis of Parkinson's disease patients.<br>• Modulates genes expression of proteins involved in autophagy-lysosomal pathway.	Phase II<br>(2018-2021)<br>(2014-2019)	[7]<br>NCT03466723<br>NCT02283073
	Resveratrol and derivatives	• Induces AMPK-dependent autophagy and α-syn clearance.<br>• Exhibits neuroprotection by reducing inflammation and enhancing pAkt/Akt ratio.	Preclinical: MPTP mice	[8][9]
c-ABL	Nilotinib	• Increases α-syn clearance.<br>• Improves motor function.<br>• Reduces oxidative stress.<br>• Protects DA neurons.	Phase II<br>2017-2019<br>Phase II<br>2017-2020	[10]<br>NCT03205488<br><br>NCT02954978
GCase	Ambroxol	• Raises the levels of the enzyme β-glucocerebrosidase.	Phase II<br>(2015-2021)<br>Phase I<br>(2016-2018)<br>Phase II<br>(2020-2023)	[11] [12]<br>NCT02914366<br><br>NCT02941822<br><br>NCT04388969
	Quetiapine*	• Leads to activation of GCase and reduction of α-synuclein.	Phase IV<br>(2022-2025)	[13]<br>NCT04373317
Glucacon-like peptide-1(GLP-1) receptor	Exenatide*	• Slows down or stops the degeneration of nerve cells<br>• Reduced anti-inflammatory response of macrophages likely to PKA and AKT activation	Phase III<br>(2020-2024)<br>phase II<br>(2020-2022)<br>phase II<br>(2020-2023)<br>Preclinical: Macrophages culture from diabetes patients	[14]<br>NCT04232969<br><br>NCT04305002<br><br>NCT04154072<br>[15]
	Liraglutide	• Alleviates the chronic inflammation<br>• Increase beclin1, atg7, LC3 and P62 expression	Phase II<br>(2017-2022)	NCT02953665<br>[16] [17]

		• Supress the expression of AKT and mTOR.		[18]
GLP1R + GIPR	DA-CH5	• Reduces microglia and astrocyte activation.<br>• Improves mitochondrial activity and increases beclin1 and LC3 expression.	Preclinical: MPTP mice	[19]
Glucacon-like peptide-2 (GLP-2) receptor	GLP-2 analogue	• Attenuates neuroinflammation.<br>• Improves motor function.<br>• Neuroprotection.<br>• Protects against the increase of autophagy.	Preclinical: MPP+ in SH-SY5Y and Neuro-2a cells	[20]
Glucosyl-ceramide (GS) synthase	Venglustat	• Reduces accumulation of glucosylceramide by preventing its synthesis by inhibiting the enzyme.	Phase II 2016-2021	[21] NCT02906020
GM1	LIGA-20	• Promotes the recovery of dopamine levels in the striatum	Preclinical: MPTP mouse PD model	[22]
IMPase	Valproate*	• Increases LC3-II levels, decreases p62 expression and Mtor phosphorylation.<br>• Reduces mitochondrial membrane potential<br>• Enhances cell viability	Phase IV (2016-2021)	[23] [24] NCT02670161
JNK	Sargramostim (GM-CSF Leukine)	• Protects dopaminergic neurons, resulting in the reduce reduction of motor symptoms	Phase I 2019-2022 2011-2025	[25] [26] NCT03790670 NCT02018406
LRRK2	BIIB094	• LRRK2 RNA.	Phase I (2019-2023)	[27] NCT03976349
	DNL151	• Inhibits LRRK2 kinase.	Phase Ib (2019-2020)	[28] NCT04056689
Miro1	Miro1 reducer	• Depolarizes mitochondria to facilitate their clearance via mitophagy.<br>• Improves locomotory functions	Preclinical: Patients-derived neurons and *Drosophila*	[29]
miRNAs	miRNA biomarkers	• Regulates gene expression by either degrading or making the targeted mRNAs	2018-2019 2014-2019 2017-2020	NCT03466723 NCT02283073 NCT03217396
mTORC1	Ibidulast	• Inhibits mTOR complex 1.<br>• Promotes the nuclear translocation of transcription factor EB, inducing autophagy.	Preclinical: Mouse embryonic fibroblast (MEF) cells	[30]
	Icariin	• Reduces phosphorylation of mTOR<br>• Activates NRF2 (regulator of macroautophagy genes)	Preclinical: Rotenone rats, PC12 cells and 6-OHDA mice	[31] [32][33]
	Sirolimus (Rapamycin)	• Inhibits the activation of the mTOR pathway, thereby protecting against loss of dopaminergic neurons<br>• Provides behavioral improvements	Phase 1/2a (2020-2027)	[34] NCT04127578
	Sirolimus + RTB101	Inhibits mTOR complex 1, which contributes to the decline of neurologic function.	Phase 1b/2a (2019-2020)	[34] [35] ACTRN12619000372189
NLRP3	Inzomelid	Inhibits inflammasomes containing NLRP3 and achieves neuroprotection.	Phase I (2019-2020)	[36], [37] NCT04015076

PI3K	Rhynchophyllline	• Improves motor function<br>• Inhibits MPP+ triggered neurotoxicity<br>• Activates the PI3K/Akt signaling pathway	Preclinical: MPTP mice/ PC12cells	[38]
	Niacin/ Niacinamide	• Lysosomal degradation of protein<br>• Activates a cellular protein called SIRT1<br>• Activates PI3K/Akt cascade	Phase II (2019-2021) (2020-2024)	NCT03808961 NCT03815916 [39] [40]
	Kukoamine A	• Inhibits iron accumulation<br>• Inhibits of trypanothione reductase<br>• Upregulates the expression of ferroportin1 and hephaestin<br>• Phosphorylates PI3K/Akt/GSK-3β signaling pathway proteins.	Preclinical: 6-OHDA PC12 cell culture	[41][42]
PINK1	Celastrol	• Enhances mitophagy (increase PINK1/DJ1).<br>• Inhibits IKK-NF-κB signalling.	Preclinical: MPTP mouse	[43] [44]
TRPML1	Clioquinol	• Functions as a zinc and copper chelator.<br>• Increase Ca^{+2} lysosomal release.	Preclinical: Rat/MTPT monkeys	[45][46]
	ML-SA1	• Protects motor neurons from L-BMAA neurotoxicity by promoting autophagic clearance.	Preclinical: DA neurons in human PD patients	[47][48]
	Rifampicin	• Inhibits rotenone-induced microglia inflammation by enhancing autophagy	Preclinical: Human microglia cells	[49]

Table1. Autophagy-related potential drugs for the treatment of Parkinson's diseases. The table represents the list of pharmacologically and naturally occurring agents designed/discovered that target different pathways that either enhance or inhibit autophagy from treating Parkinsons' disease. Some of the agents are currently ongoing clinical trials at different stages and some are in the experimental phases in different model organisms. Compounds marked with (*) are not cited in the text but they are included because they are in clinical trial phase III/IV. N/A means not applicable.

[1] W. Liu *et al.*, “Regular aerobic exercise-alleviated dysregulation of CAMKIIa carbonylation to mitigate parkinsonism via homeostasis of apoptosis with autophagy,” *J. Neuropathol. Exp. Neurol.*, vol. 79, no. 1, pp. 46–61, 2020, doi: 10.1093/jnen/nlz106.

[2] E. Seweryn, A. Ziała, and A. Gamian, “Health-Promoting of Polysaccharides Extracted from Ganoderma lucidum,” *Nutr. 2021, Vol. 13, Page 2725*, vol. 13, no. 8, p. 2725, Aug. 2021, doi: 10.3390/NU13082725.

[3] N. G. Norwitz, D. J. Dearlove, M. Lu, K. Clarke, H. Dawes, and M. T. Hu, “A Ketone Ester Drink Enhances Endurance Exercise Performance in Parkinson’s Disease,” *Front. Neurosci.*, vol. 14, no. September, pp. 1–11, 2020, doi: 10.3389/fnins.2020.584130.

[4] Z. Kovács, B. Brunner, and C. Ari, “Beneficial effects of exogenous ketogenic supplements on aging processes and age-related neurodegenerative diseases,” *Nutrients*, vol. 13, no. 7, pp. 1–35, 2021, doi: 10.3390/nu13072197.

[5] S. H. El-Ghaiesh *et al.*, “Metformin Protects From Rotenone–Induced Nigrostriatal Neuronal Death in Adult Mice by Activating AMPK-FOXO3 Signaling and Mitigation of Angiogenesis,” *Front. Mol. Neurosci.*, vol. 13, no. June, pp. 1–14, 2020, doi: 10.3389/fnmol.2020.00084.

[6] N. Katila, S. Bhurtel, P. H. Park, J. T. Hong, and D. Y. Choi, "Activation of AMPK/aPKCζ/CREB pathway by metformin is associated with upregulation of GDNF and dopamine," *Biochem. Pharmacol.*, vol. 180, no. August, p. 114193, 2020, doi: 10.1016/j.bcp.2020.114193.

[7] E. Angelopoulou, Y. N. Paudel, and C. Piperi, "miR-124 and Parkinson's disease: A biomarker with therapeutic potential," *Pharmacol. Res.*, vol. 150, no. July, p. 104515, 2019, doi: 10.1016/j.phrs.2019.104515.

[8] Q. Liu *et al.*, "Resveratrol synergizes with low doses of L-DOPA to improve MPTP-induced Parkinson disease in mice," *Behav. Brain Res.*, vol. 367, no. February, pp. 10–18, 2019, doi: 10.1016/j.bbr.2019.03.043.

[9] B. D. Arbo *et al.*, "Resveratrol Derivatives as Potential Treatments for Alzheimer's and Parkinson's Disease," *Front. Aging Neurosci.*, vol. 12, no. April, pp. 1–15, 2020, doi: 10.3389/fnagi.2020.00103.

[10] "Nilotinib for Parkinson's Disease - Parkinson's News Today." .

[11] C. R. A. Silveira *et al.*, "Ambroxol as a novel disease-modifying treatment for Parkinson's disease dementia: Protocol for a single-centre, randomized, double-blind, placebo-controlled trial," *BMC Neurol.*, vol. 19, no. 1, pp. 1–10, 2019, doi: 10.1186/s12883-019-1252-3.

[12] S. Mullin *et al.*, "Ambroxol for the Treatment of Patients with Parkinson Disease with and without Glucocerebrosidase Gene Mutations: A Nonrandomized, Noncontrolled Trial," *JAMA Neurol.*, pp. 1–8, 2020, doi: 10.1001/jamaneurol.2019.4611.

[13] L. F. Burbulla *et al.*, "Direct targeting of wild-type glucocerebrosidase by antipsychotic quetiapine improves pathogenic phenotypes in Parkinson's disease models," *JCI Insight*, vol. 6, no. 19, pp. 1–8, 2021, doi: 10.1172/jci.insight.148649.

[14] C. S. Storm *et al.*, "Predicting the efficacy of exenatide in Parkinson's disease using genetics – a Mendelian randomization study," *medRxiv*, p. 2020.10.20.20215855, Oct. 2020, doi: 10.1101/2020.10.20.20215855.

[15] Ł. Bułdak *et al.*, "Exenatide (a GLP-1 agonist) expresses anti-inflammatory properties in cultured human monocytes/macrophages in a protein kinase A and B/Akt manner," *Pharmacol. Reports*, vol. 68, no. 2, pp. 329–337, 2016, doi: 10.1016/j.pharep.2015.10.008.

[16] K. McFarthing, D. Larson, and T. Simuni, "Clinical Trial Highlights - GLP-1 agonists," *J. Parkinsons. Dis.*, vol. 10, no. 2, pp. 355–368, 2020, doi: 10.3233/JPD-200002.

[17] L. Zhang, L. Zhang, L. Li, and C. Hölscher, "Semaglutide is neuroprotective and reduces α-synuclein levels in the chronic MPTP mouse model of Parkinson's disease," *J. Parkinsons. Dis.*, vol. 9, no. 1, pp. 157–171, 2019, doi: 10.3233/JPD-181503.

[18] T. T. Liao, L. B. Zhao, H. Liu, R. L. He, Y. Q. Wang, and J. Li, "Liraglutide protects from renal damage via Akt-mTOR pathway in rats with diabetic kidney disease," *Eur. Rev. Med. Pharmacol. Sci.*, vol. 23, no. 3, pp. 117–125, 2019, doi: 10.26355/EURREV_201908_18638.

[19] L. Zhang *et al.*, "The Novel Dual GLP-1/GIP Receptor Agonist DA-CH5 Is Superior to Single GLP-1 Receptor Agonists in the MPTP Model of Parkinson's Disease," *J. Parkinsons. Dis.*, vol. 10, no. 2, pp. 523–542, 2020, doi: 10.3233/JPD-191768.

[20] Y. Su *et al.*, "A GLP-2 Analogue Protects SH-SY5Y and Neuro-2a Cells against Mitochondrial Damage, Autophagy Impairments and Apoptosis in a Parkinson Model," *Drug Res. (Stuttg).*, vol. 71, no. 1, pp. 43–50, 2021, doi: 10.1055/a-1266-3263.

[21] S. Mullin *et al.*, "Ambroxol for the Treatment of Patients With Parkinson Disease With and Without Glucocerebrosidase Gene Mutations: A Nonrandomized, Noncontrolled Trial," *JAMA Neurol.*, vol. 77, no. 4, pp. 427–434, Apr. 2020, doi: 10.1001/JAMANEUROL.2019.4611.

[22] I. Alecu and S. A. L. Bennett, "Dysregulated lipid metabolism and its role in α-synucleinopathy in Parkinson's

disease," *Front. Neurosci.*, vol. 13, no. APR, p. 328, 2019, doi: 10.3389/FNINS.2019.00328/BIBTEX.

[23] A. Muralidharan, J. Rahman, D. Banerjee, A. R. Hakim Mohammed, and B. H. Malik, "Parkinsonism: A Rare Adverse Effect of Valproic Acid," *Cureus*, vol. 12, no. 6, Jun. 2020, doi: 10.7759/CUREUS.8782.

[24] E. H. Jang, J. H. Lee, and S. A. Kim, "Acute valproate exposure induces mitochondrial biogenesis and autophagy with foxo3a modulation in sh-sy5y cells," *Cells*, vol. 10, no. 10, 2021, doi: 10.3390/cells10102522.

[25] H. E. Gendelman *et al.*, "Evaluation of the safety and immunomodulatory effects of sargramostim in a randomized, double-blind phase 1 clinical Parkinson's disease trial," *NPJ Park. Dis.*, vol. 3, no. 1, Dec. 2017, doi: 10.1038/S41531-017-0013-5.

[26] Y. Zhang, M. J. Morgan, K. Chen, S. Choksi, and Z. G. Liu, "Induction of autophagy is essential for monocyte-macrophage differentiation," *Blood*, vol. 119, no. 12, pp. 2895–2905, 2012, doi: 10.1182/blood-2011-08-372383.

[27] K. McFarthing, S. Buff, G. Rafaloff, T. Dominey, R. K. Wyse, and S. R. W. Stott, "Parkinson's Disease Drug Therapies in the Clinical Trial Pipeline: 2020," *J. Parkinsons. Dis.*, vol. 10, no. 3, p. 757, 2020, doi: 10.3233/JPD-202128.

[28] "LRRK2 Inhibition by BIIB122 / DNL151: Safety, Tolerability, Pharmacokinetics, and Pharmacodynamics in Ph 1 Healthy Volunteer and Ph 1B Parkinson's Disease Trials - MDS Abstracts." .

[29] C. H. Hsieh *et al.*, "Miro1 Marks Parkinson's Disease Subset and Miro1 Reducer Rescues Neuron Loss in Parkinson's Models," *Cell Metab.*, vol. 30, no. 6, pp. 1131-1140.e7, 2019, doi: 10.1016/j.cmet.2019.08.023.

[30] Y. Chen, H. Wang, Z. Ying, and Q. Gao, "Ibudilast enhances the clearance of SOD1 and TDP-43 aggregates through TFEB-mediated autophagy and lysosomal biogenesis: The new molecular mechanism of ibudilast and its implication for neuroprotective therapy," *Biochem. Biophys. Res. Commun.*, vol. 526, no. 1, pp. 231–238, 2020, doi: 10.1016/j.bbrc.2020.03.051.

[31] R. Zeng *et al.*, "Icariin-mediated activation of autophagy confers protective effect on rotenone induced neurotoxicity in vivo and in vitro," *Toxicol. Reports*, vol. 6, no. June, pp. 637–644, 2019, doi: 10.1016/j.toxrep.2019.06.014.

[32] B. Zhang *et al.*, "Icariin attenuates neuroinflammation and exerts dopamine neuroprotection via an Nrf2-dependent manner," *J. Neuroinflammation*, vol. 16, no. 1, pp. 1–11, 2019, doi: 10.1186/s12974-019-1472-x.

[33] S. Wang *et al.*, "Icariin, an up-and-coming bioactive compound against neurological diseases: Network pharmacology-based study and literature review," *Drug Des. Devel. Ther.*, vol. 15, no. August, pp. 3619–3641, 2021, doi: 10.2147/DDDT.S310686.

[34] BioSpace, "resTORbio Announces Delay of its Ongoing Phase 1b/2a trial of RTB101 in Patients with Parkinson's Disease Due to COVID-19 Level 4 Alert in New Zealand," 2020. https://www.biospace.com/article/releases/restorbio-announces-delay-of-its-ongoing-phase-1b-2a-trial-of-rtb101-in-patients-with-parkinson-s-disease-due-to-covid-19-level-4-alert-in-new-zealand/.

[35] "Pipeline - resTORbio." .

[36] L. Y. El-Sharkawy, D. Brough, and S. Freeman, "Inhibiting the NLRP3 Inflammasome," *Mol. 2020, Vol. 25, Page 5533*, vol. 25, no. 23, p. 5533, Nov. 2020, doi: 10.3390/MOLECULES25235533.

[37] D. Tejera *et al.*, " Systemic inflammation impairs microglial Aβ clearance through NLRP 3 inflammasome ," *EMBO J.*, vol. 38, no. 17, Sep. 2019, doi: 10.15252/embj.2018101064.

[38] M. Zheng *et al.*, "Protection by rhynchophylline against MPTP/MPP+-induced neurotoxicity via regulating PI3K/Akt pathway," *J. Ethnopharmacol.*, vol. 268, p. 113568, 2021, doi: 10.1016/j.jep.2020.113568.

[39] R. Chong, C. Wakade, M. Seamon, B. Giri, J. Morgan, and S. Purohit, "Niacin Enhancement for Parkinson's Disease: An Effectiveness Trial," *Front. Aging Neurosci.*, vol. 13, p. 210, Jun. 2021, doi: 10.3389/FNAGI.2021.667032/BIBTEX.

[40] S. W. Kim *et al.*, "Niacin alleviates TRAIL-mediated colon cancer cell death via autophagy flux activation," *Oncotarget*, vol. 7, no. 4, pp. 4356–4368, 2016, doi: 10.18632/oncotarget.5374.

[41] X. Li, X. wen Jiang, H. xiao Chu, Q. chun Zhao, H. wei Ding, and C. hong Cai, "Neuroprotective effects of kukoamine A on 6-OHDA-induced Parkinson's model through apoptosis and iron accumulation inhibition," *Chinese Herb. Med.*, vol. 13, no. 1, pp. 105–115, 2021, doi: 10.1016/j.chmed.2020.12.004.

[42] Y. Yang *et al.*, "Kukoamine A Protects against NMDA-Induced Neurotoxicity Accompanied with Down-Regulation of GluN2B-Containing NMDA Receptors and Phosphorylation of PI3K/Akt/GSK-3β Signaling Pathway in Cultured Primary Cortical Neurons," *Neurochem. Res.*, vol. 45, no. 11, pp. 2703–2711, 2020, doi: 10.1007/s11064-020-03114-y.

[43] M. W. Lin, C. C. Lin, Y. H. Chen, H. Bin Yang, and S. Y. Hung, "Celastrol inhibits dopaminergic neuronal death of Parkinson'S disease through activating mitophagy," *Antioxidants*, vol. 9, no. 1, 2019, doi: 10.3390/antiox9010037.

[44] C. Zhang *et al.*, "The plant triterpenoid celastrol blocks PINK1-dependent mitophagy by disrupting PINK1's association with the mitochondrial protein TOM20," *J. Biol. Chem.*, vol. 294, no. 18, pp. 7472–7487, 2019, doi: 10.1074/jbc.RA118.006506.

[45] R. Wallings, N. Connor-Robson, and R. Wade-Martins, "LRRK2 interacts with the vacuolar-type H+-ATPase pump a1 subunit to regulate lysosomal function," *Hum. Mol. Genet.*, vol. 28, no. 16, pp. 2696–2710, 2019, doi: 10.1093/hmg/ddz088.

[46] L. Shi *et al.*, "Clioquinol improves motor and non-motor deficits in MPTP-induced monkey model of Parkinson ' s disease through AKT / mTOR pathway," vol. 12, no. 10, pp. 9515–9533, 2020.

[47] T. Tsunemi *et al.*, "Increased Lysosomal Exocytosis Induced by Lysosomal Ca2+ Channel Agonists Protects Human Dopaminergic Neurons from α-Synuclein Toxicity," *J. Neurosci.*, vol. 39, no. 29, pp. 5760–5772, 2019, doi: 10.1523/JNEUROSCI.3085-18.2019.

[48] V. Tedeschi, T. Petrozziello, M. J. Sisalli, F. Boscia, L. M. T. Canzoniero, and A. Secondo, "The activation of Mucolipin TRP channel 1 (TRPML1) protects motor neurons from L-BMAA neurotoxicity by promoting autophagic clearance," *Sci. Rep.*, vol. 9, no. 1, pp. 1–11, 2019, doi: 10.1038/s41598-019-46708-5.

[49] Y. Liang *et al.*, "Rifampicin attenuates rotenone-treated microglia inflammation via improving lysosomal function," *Toxicol. Vitr.*, vol. 63, p. 104690, 2020, doi: 10.1016/j.tiv.2019.104690.
